# Feasibility of ultrashort echo time quantitative susceptibility mapping with a 3D cones trajectory in the human brain

**DOI:** 10.3389/fnins.2022.1033801

**Published:** 2022-11-07

**Authors:** Hyungseok Jang, Sam Sedaghat, Jiyo S. Athertya, Dina Moazamian, Michael Carl, Yajun Ma, Xing Lu, Alicia Ji, Eric Y. Chang, Jiang Du

**Affiliations:** ^1^Department of Radiology, University of California, San Diego, San Diego, CA, United States; ^2^GE HealthCare, San Diego, CA, United States; ^3^Radiology Service, Veterans Affairs (VA) San Diego Healthcare System, San Diego, CA, United States; ^4^Department of Bioengineering, University of California, San Diego, San Diego, CA, United States

**Keywords:** magnetic susceptibility, brain, quantitative susceptibility mapping, QSM, MRI, UTE, cones, spiral

## Abstract

**Purpose:**

Quantitative susceptibility mapping (QSM) has surfaced as a promising non-invasive quantitative biomarker that provides information about tissue composition and microenvironment. Recently, ultrashort echo time quantitative susceptibility mapping (UTE-QSM) has been investigated to achieve QSM of short T2 tissues. As the feasibility of UTE-QSM has not been demonstrated in the brain, the goal of this study was to develop a UTE-QSM with an efficient 3D cones trajectory and validate it in the human brain.

**Materials and methods:**

An ultrashort echo time (UTE) cones sequence was implemented in a 3T clinical MRI scanner. Six images were acquired within a single acquisition, including UTE and gradient recalled echo (GRE) images. To achieve QSM, a morphology-enabled dipole inversion (MEDI) algorithm was incorporated, which utilizes both magnitude and phase images. Three fresh cadaveric human brains were scanned using the 3D cones trajectory with eight stretching factors (SFs) ranging from 1.0 to 1.7. In addition, five healthy volunteers were recruited and underwent UTE-QSM to demonstrate the feasibility *in vivo*. The acquired data were processed with the MEDI-QSM pipeline.

**Results:**

The susceptibility maps estimated by UTE-QSM showed reliable tissue contrast. In the *ex vivo* experiment, high correlations were found between the baseline (SF of 1.0) and SFs from 1.1 to 1.7 with Pearson’s correlations of 0.9983, 0.9968, 0.9959, 0.9960, 0.9954, 0.9943, and 0.9879, respectively (all *p*-values < 0.05). In the *in vivo* experiment, the measured QSM values in cortical gray matter, juxtacortical white matter, corpus callosum, caudate, and putamen were 25.4 ± 4.0, −21.8 ± 3.2, −22.6 ± 10.0, 77.5 ± 18.8, and 53.8 ± 7.1 ppb, consistent with the values reported in the literature.

**Conclusion:**

Ultrashort echo time quantitative susceptibility mapping enables direct estimation of the magnetic susceptibility in the brain with a dramatically reduced total scan time by use of a stretched 3D cones trajectory. This technique provides a new biomarker for susceptibility mapping in the *in vivo* brain.

## Introduction

Magnetic susceptibility is a fundamental property of a substance, which refers to the natural response to the applied magnetic field. In an MRI system, tissues placed in the B0 field create their local dipole field and disturbing the external magnetic field. Therefore, tissues with strong magnetic susceptibilities may result in degradation of the MR image due to the disturbed B0 field, resulting in artifactual signal dropout/enhancement or spatial distortion. To alleviate the B0 field distortion, various shimming techniques have been incorporated into modern clinical MR systems, which compensate for the field inhomogeneity by superimposing additional linear or higher-order magnetic fields (Vannesjo et al., 2014).

On the other hand, the susceptibility effect in MRI has been harnessed to achieve signal weighting (or contrast) specific to the chemical composition of the targeted tissues. Conventional susceptibility weighted imaging (SWI) (Hodel et al., 2013; Barbosa et al., 2015a) and blood oxygenation level-dependent (BOLD) (Chen and Pike, 2009; Le Bihan, 2012) imaging are clinically used imaging techniques based on susceptibility effects. In SWI, both magnitude and phase data are acquired with high T2* weighting (i.e., long echo time) to generate susceptibility-weighted contrast that is sensitive to both positive (i.e., paramagnetic) and negative (i.e., diamagnetic) susceptibility sources. SWI has been used to detect microhemorrhage, hemosiderin, and calcification in various body parts. BOLD imaging is a standard method for functional MRI based on blood oxygenation in the brain. The BOLD signal is also heavily T2* weighted, providing MRI signal contrast specific to deoxygenated hemoglobin in blood, which reflects brain activity.

Over the past decade, quantitative susceptibility mapping (QSM) has emerged as a promising quantitative MR imaging technique to assess the tissue chemical composition and microenvironment (Deistung et al., 2013; Langkammer et al., 2013; Wang and Liu, 2015; Acosta-Cabronero et al., 2016; Liu et al., 2018). QSM estimates tissue susceptibility values by measuring the distorted B0 field based on the phase evolution in a free induction decay and applying advanced reconstruction algorithms, including a phase unwrapping, a background field removal, and a dipole inversion. In literatures, many QSM methods have been proposed, such as the calculation of susceptibility through multiple orientation sampling (COSMOS) (Liu et al., 2009), morphology-enabled dipole inversion (MEDI) (Liu et al., 2012), improved sparse linear equation and least-squares (iLSQR) algorithm (Straub et al., 2017), and streaking artifact reduction for QSM (STAR-QSM) (Wei et al., 2015). Most are based on Cartesian gradient recalled echo (GRE) sequences.

To overcome the limitation of GRE-based QSM, which cannot directly image short T2 tissues such as bone, tendon, ligament, meniscus, and hemosiderin, ultrashort echo time (UTE) based QSM has been recently proposed (Dimov et al., 2018; Jerban et al., 2019; Lu et al., 2019; Jang et al., 2020). UTE imaging is typically based on center-out non-Cartesian radial k-space trajectories with the omitted rewinding gradient to shorten the minimum echo time (TE). UTE-based QSM (UTE-QSM) has shown its feasibility for the assessment of bone mineral density (BMD) in osteoporosis (Dimov et al., 2018; Jerban et al., 2019) and hemosiderin in hemophilic arthropathy (Jang et al., 2020). As non-Cartesian UTE sequences have not been explored in the brain QSM yet, this study evaluated the feasibility of UTE-QSM based on a 3D spiral cones trajectory in the human brain. The UTE-QSM was tested on three cadaveric human brains and five healthy volunteers.

## Materials and methods

### Pulse sequence

**FIGURE 1:**
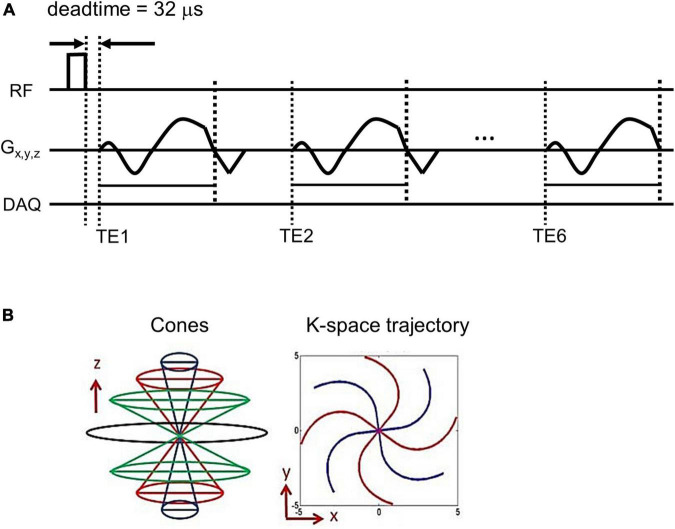
Pulse sequence diagram. An ultrashort echo time quantitative susceptibility mapping (UTE-QSM) sequence **(A)** based on the efficient 3D cones trajectory **(B)** was implemented to acquire images at six different echo times for QSM processing (DAQ: Data acquisition).

A 3D UTE cone sequence was implemented on a 3T clinical MRI scanner (MR 750, GE Healthcare, Milwaukee, WI, USA). Figure 1A shows the pulse sequence diagram of the 3D UTE cones sequence used in the UTE-QSM study. To achieve an ultrashort TE of 32 μs, data readout was performed immediately after RF deadtime, based on a k-space-center-to-out acquisition scheme. In this study, a spiral cones readout trajectory was adopted to allow more efficient encoding of the 3D k-space (Figure 1B). In the 3D cones imaging, spiral arms were first generated with different polar angles with respect to the kz axis and rotated in the kx-ky plane to cover the 3D spherical k-space. The length of the spiral arm can be adjusted, where fewer spokes are required with a more extended (or stretched) spiral arm (i.e., shorter scan time) and vice versa. The relative stretching factor (SF) was defined as the ratio between the readout durations (or length of spiral arms) with and without stretching. A higher SF yields more efficient coverage of the k-space with a smaller number of spokes.

Using the UTE cones sequence, a total of six echoes were achieved within a single acquisition based on a fly-back UTE-GRE echo train scheme. Note that the shape of the readout gradient is maintained the same for all echoes.

### Imaging experiment

To show the feasibility, an *ex vivo* experiment was performed by scanning three fresh cadaveric human brain samples from donors without any history of previous neurological diseases (82-year-old female, 70-year-old female, and 67-year-old male). The brains were harvested post-mortem and underwent MRI on a 3T clinical MRI scanner (MR750, GE Healthcare) using a clinical 8-channel receive-only head coil. The imaging parameters of UTE cones sequence were as follows: TR = 50 ms, TE = 0.032, 4.4, 8.8, 13.2, 17.6, and 22 ms, flip angle (FA) = 20 degree, field of view (FOV) = 220 × 220 × 80 mm^3^, matrix = 256 × 256 × 80, and readout bandwidth = 166.6 kHz. The scan was repeated with eight different SFs of the 3D cones spiral arms: 1.0, 1.1, 1.2, 1.3, 1.4, 1.5, 1.6, and 1.7 with the corresponding scan times of 23.1, 20.5, 18.3, 16.8, 15.3, 14.3, 13.2, and 12.4 min, respectively.

An *in vivo* experiment was performed on five healthy volunteers (two females and three males, 37.8 ± 7.5 years old) under the approval of the human research protections program (HRPP) of the University of California, San Diego. Written consent was obtained from each participant before MR examination. The volunteers underwent the same 3D UTE cones brain imaging protocol as used for the *ex vivo* experiment except for the following different parameters: TR = 30 ms, FA = 20 degree, FOV = 220 × 220 × 160 mm^3^, matrix = 220 × 220 × 80, SF = 1.7, and scan time = 10.8 min.

### Data processing

All data processing was done in using Matlab R2017b (MathWorks, Natick, MA, USA). The images were reconstructed using a non-uniform fast Fourier transform (NuFFT) algorithm (Fessler, 2007). Complex data acquired by multiple phased array coil elements (eight channels in this study) were combined using coil sensitivity information retrieved by singular value decomposition (Walsh et al., 2000). Subsequently, the resultant complex MR images at different TEs were processed with a QSM reconstruction pipeline established based on a MEDI toolbox.

In the MEDI-based QSM framework, a B0 field inhomogeneity map was first estimated using a least square fitting algorithm in which the slope of the fitted phase data yields the off-resonant frequency. The estimated B0 field map was processed with projection onto a dipole field (PDF) algorithm to remove the background field and obtain a local tissue dipole field (Liu et al., 2011a). The local field map was processed with dipole inversion using the MEDI algorithm with a Lagrange multiplier of 1000.

In the *ex vivo* experiment, the mean susceptibility values were calculated in 36 manually selected regions of interest (ROIs) from all three cadaveric brains to demonstrate the impact of stretched 3D cones spiral arms in UTE-QSM. The ROIs were taken from the regions of juxtacortical white matter (*n* = 12), cortical gray matter (*n* = 12), and the boundaries between white matter and gray matter (*n* = 12). Then, Pearson’s correlations (R) were calculated between UTE-QSM with no stretching (i.e., SF = 1.0) and with stretching (i.e., SF = 1.1, 1.2, 1.3, 1.4, 1.5, 1.6, or 1.7). In the *in vivo* experiment, the mean and standard deviation of the susceptibility were calculated from the drawn ROIs in the cortical gray matter, juxtacortical white matter, corpus callosum, caudate, and putamen. The ROIs were manually drawn by a researcher with 10 years of experience in MRI research under the supervision of a neuroradiologist with 8 years of experience.

## Results

### *Ex vivo* experiment

**FIGURE 2:**
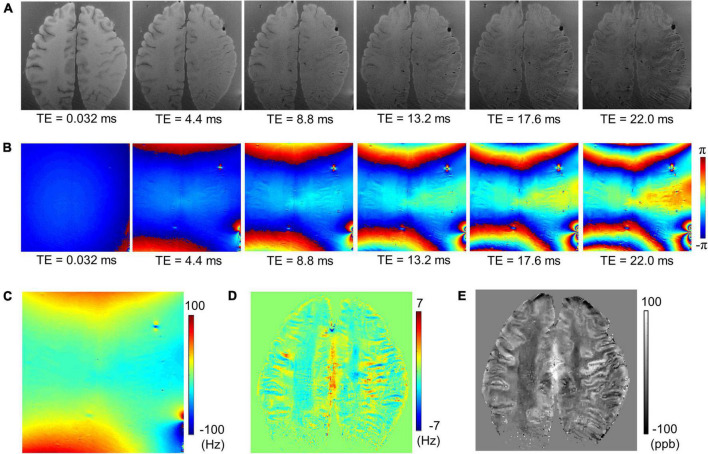
Ultrashort echo time quantitative susceptibility mapping (UTE-QSM) with a cadaveric brain (82-year-old female donor). **(A)** Magnitude images, **(B)** phase images, **(C)** the total field map, **(D)** the local field map after background field removal, and **(E)** the resultant susceptibility map.

Figure 2 shows an example of UTE-QSM processing of a representative cadaveric brain (82-year-old female donor). Figures 2A,B show the magnitude and phase of the input images acquired at six different TEs. Figures 2C–E show the total field map acquired from the phase images, the local field map obtained using the PDF algorithm, and the resultant UTE-QSM susceptibility map, respectively.

**FIGURE 3:**
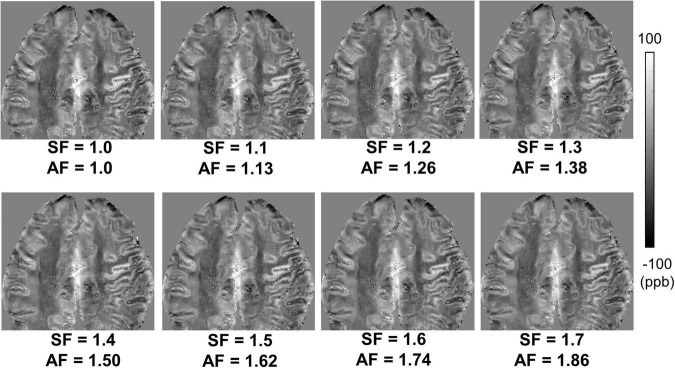
*Ex vivo* ultrashort echo time quantitative susceptibility mapping (UTE-QSM) in a representative cadaveric brain (82-year-old female donor) with various stretching factors (SFs) to achieve different acceleration factors (AFs). The stretched cones trajectories dramatically reduced the total scan time (e.g., an AF of 1.86 with an SF of 1.7) without noticeable degradation in QSM.

**FIGURE 4:**
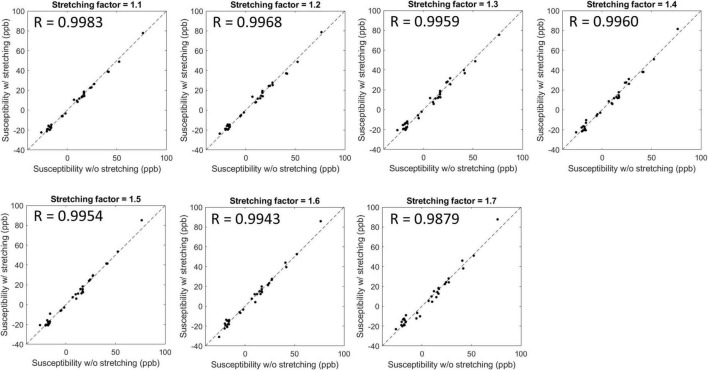
Scatter plots and Pearson’s correlation between susceptibility values estimated without and with stretched cones trajectories. The estimated susceptibility showed a high linear correlation with a stretching factor up to 1.7 with *R* > 0.98 and *p*-value < 0.05.

Figure 3 shows susceptibility maps obtained with various SFs of cones trajectory. The estimated susceptibility maps exhibited tissue contrast with clear discrimination between diamagnetic white matter and paramagnetic gray matter. There was no dramatic visual difference found between the susceptibility maps without and with stretched cones trajectory. Figure 4 shows the scatter plots of mean susceptibility values (with and without stretching). As shown in the plots, very high correlations were found between SF of 1.0 and SFs from 1.1 to 1.7 with R of 0.9983, 0.9968, 0.9959, 0.9960, 0.9954, 0.9943, and 0.9879, respectively (all *p* < 0.05).

### *In vivo* experiment

**FIGURE 5:**
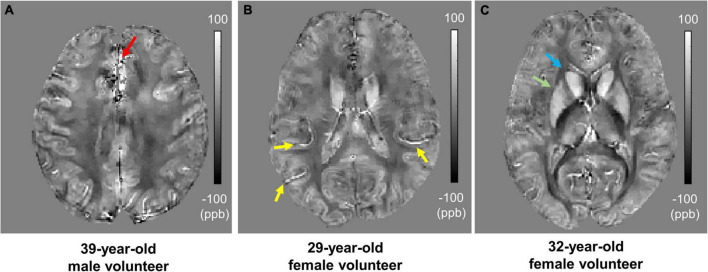
*In vivo* ultrashort echo time quantitative susceptibility mapping (UTE-QSM) of three representative healthy volunteers. **(A)** 39-year-old male, **(B)** 29-year-old female, and **(C)** 32-year-old female. The estimated susceptibility maps show a clear contrast between different tissues including the white matter, gray matter, vessels (yellow arrows), caudate (blue arrow), and putamen (green arrow). Artifactually elevated susceptibility was observed in the region indicated by the red arrow which is presumably due to streaking artifacts or imperfection in UTE-QSM.

In the *in vivo* experiment, UTE-QSM with cones trajectory yielded susceptibility maps without noticeable artifacts in the brains of all participants. Figure 5 shows the resultant susceptibility maps from three healthy representative volunteers in axial views. The estimated susceptibility maps depict the different brain tissues, including the white matter, gray matter, vessels (yellow arrows), caudate (blue arrow), and putamen (green arrow). The measured susceptibility values in cortical gray matter, juxtacortical white matter, corpus callosum, caudate, and putamen were 25.4 ± 4.0, −21.8 ± 3.2, −22.6 ± 10.0, 77.5 ± 18.8, and 53.8 ± 7.1 ppb, respectively, which correspond well with the values reported in the literature (Li et al., 2011; Liu et al., 2012; Acosta-Cabronero et al., 2016).

Although no significant artifacts were visible in the estimated susceptibility maps, artifactually elevated susceptibility was observed (red arrow) which is presumably due to streaking artifact propagated from neighboring tissues with strong susceptibility such as air in sinuses and cavities or due to any imperfection in UTE-QSM including eddy current effect and intra-scan motion.

## Discussion

This study demonstrated the feasibility of UTE-QSM with a 3D cones trajectory in the human brain for the first time. The proposed UTE-QSM achieved reasonable susceptibility maps in *ex vivo* and *in vivo* brains. The efficacy of UTE imaging and the contribution of ultrashort TE in QSM has been demonstrated in previous studies (Dimov et al., 2018; Lu et al., 2018; Jerban et al., 2019; Jang et al., 2020). This preliminary study focused on developing and optimizing 3D UTE-QSM sequence in the human brain. The susceptibility values estimated with UTE-QSM were within the reasonable range based on the values reported in the literature. For example, Liu et al. (2011b) reported 46–53 ppb in gray matter, 82–86 ppb in putamen, and 80–89 ppb in caudate. Langkammer et al. (2013) reported 50 ppb in caudate and 53 ppb in putamen. Barbosa et al. (2015b) reported 63 ppb in putamen, and Acosta-Cabronero et al. (2016) reported 33 ppb in caudate and 21 ppb in putamen. Li et al. (2011) reported 43 ppb in putamen and 19 ppb in caudate. Wisnieff et al. (2015) reported −43 to −23 ppb in white matter. Li et al. (2015) reported 49 to 51 ppb in caudate and 41 to 42 ppb in putamen. Naji et al. (2020) reported ∼-40 ppb in white matter, ∼40 ppb in putamen, and ∼30 ppb in caudate.

Our previous studies have shown the efficacy of 3D cones trajectory in UTE imaging, significantly reducing the scan time by more than 2× compared to the conventional 3D radial trajectory (Lu et al., 2019; Wan et al., 2019). The scan time can be further reduced with an additional stretching of the spiral arms in the cones imaging (Lu et al., 2019; Wan et al., 2019). We showed that UTE-QSM was achieved with a scan time of ∼11 min by using a SF of 1.7, equivalent to the acceleration factor of 1.86. In comparison, the scan time would have been over 20 min without stretching or over 40 min with radial trajectory. Despite the stretching trajectory as a promising tool, a longer readout in UTE imaging can exacerbate short T2* blurriness effects and chemical shift artifacts, which may negatively impact QSM. However, the short T2* blurriness is expected to be minimal in normal brain tissues due to the much shorter readout duration of a few milliseconds compared to the T2* of brain tissues. Moreover, chemical shift artifacts may not be a big issue in the brain, which is mainly composed of non-fatty tissues. Therefore, cones trajectory with stretching is likely to provide an effective strategy to shorten the examination time for UTE-QSM. The feasibility of stretched cones trajectory was also shown in the *ex vivo* experiment (Figures 3, 4).

In this pilot study of UTE-QSM targeting the human brain, reliable susceptibility maps showed clear contrast between different tissues (Figures 3, 5). In the *in vivo* imaging, the susceptibility values estimated with a stretched cones trajectory were within the expected range, corresponding well with the reported values in the literature (Li et al., 2011; Liu et al., 2012; Acosta-Cabronero et al., 2016). However, mild streaking artifacts were exhibited (red arrow in Figure 5A) which were presumably propagated from neighboring tissues with strong susceptibilities such as air in sinuses and cavities (i.e., oxygen as a strong paramagnetic substance). To suppress this kind of streaking artifact, a more advanced QSM algorithm similar to STAR-QSM (Wei et al., 2015) may be helpful, in which QSM processing is performed in the manner of segmented multi-stage dipole inversion to deal with both strong and weak susceptibility sources. In future studies, we will further investigate more advanced QSM techniques and incorporate them into UTE-QSM.

There are several advantages of UTE-QSM over conventional QSM. First, UTE-QSM will likely provide a more accurate assessment for calcification, hemorrhage, iron overload, and tumor vascularization in the brain, since UTE acquisition enables direct imaging of short T2 tissues with a high signal-to-noise ratio (SNR) and thereby robust mapping of susceptibilities. Second, the non-Cartesian UTE-QSM is likely to be more robust to patient motion due to the nature of radial (or cones) sampling that oversamples the central region of the k-space. Moreover, with the center-out UTE imaging, retrospective gating can be readily implemented to correct for intra-scan motion without requiring the additional acquisition of navigation data or sequence modification. Third, the inclusion of ultrashort TE images may benefit the QSM processing because of improved quality (i.e., reduced susceptibility or blooming artifacts) in both magnitude and phase images, especially in patients with accumulated iron or calcification.

On the other hand, UTE imaging may impose several challenges that should be addressed to achieve a more reliable QSM. First, eddy currents are more problematic in UTE than in Cartesian imaging due to the utilization of fast ramp sampling with a high gradient amplitude and a high slew rate. In this study, the fly-back UTE-GRE scheme was employed where the shape of the readout gradient was matched the same in all echoes. This approach can be potentially beneficial for multi-echo UTE-GRE imaging since the eddy current effect and the resultant k-space trajectory are maintained similarly between echoes. However, more active eddy current correction techniques are required to compensate for any phase errors caused by B0 and linear eddy current, further improving the accuracy of UTE-QSM (Duyn et al., 1998; Brodsky et al., 2013). Second, the long acquisition time is a potential disadvantage of UTE-QSM. We showed the feasibility of cones trajectory with a high SF to reduce the scan time in this study. More advanced acceleration techniques such as parallel imaging-based compressed sensing (PICS) (Lustig and Pauly, 2010) and deep learning-based reconstruction techniques (Zhu et al., 2018) will be able to reduce the scan time more.

This study has several limitations. First, only cadaveric brains without neurological diseases and healthy brains of volunteers were scanned in this study. The efficacy of UTE-QSM in detecting brain lesions with high susceptibility sources such as hemorrhage, calcification, and iron overload remains to be investigated. In future studies, we will apply the UTE-QSM technique to patients with neurological disorders such as Parkinson’s disease (PD), where iron overload has been implicated in the pathology and pathogenesis (Kaindlstorfer et al., 2018). The diagnostic power of UTE-QSM in PD can be demonstrated by comparing it with conventional gradient echo-based QSM techniques. However, the feasibility study on healthy volunteers is an essential first step, paving the way for future studies investigating UTE-QSM in PD and other neurological diseases. Second, although we showed that the estimated susceptibility values were within the expected range, no direct comparison was made between UTE-QSM and conventional Cartesian QSM. This will be further investigated in future studies.

## Data availability statement

The raw data supporting the conclusions of this article will be made available by the authors, without undue reservation.

## Ethics statement

The studies involving human participants were reviewed and approved by the Human Research Protections Program (HRPP) of the University of California, San Diego. The patients/participants provided their written informed consent to participate in this study.

## Author contributions

HJ, MC, XL, and YM contributed to the implementation of MRI sequences. HJ, SS, and JD designed the experiment and data processing pipeline. HJ, JA, and DM contributed to the data acquisition. HJ, SS, AJ, EC, and JD contributed to the data analysis and interpretation. All authors contributed to the manuscript revision, read, and approved the submitted version.
